# Polyphenols in Raw and Cooked Cereals/Pseudocereals/Legume Pasta and Couscous

**DOI:** 10.3390/foods6090080

**Published:** 2017-09-11

**Authors:** Marina Carcea, Valentina Narducci, Valeria Turfani, Vittoria Giannini

**Affiliations:** Research Centre for Food and Nutrition, Council for Agricultural Research and Economics (CREA-AN), Via Ardeatina 546, 00178 Rome, Italy; valentina.narducci@crea.gov.it (V.N.); valeria.turfani@crea.gov.it (V.T.); nutrivit.giannini@gmail.com (V.G.)

**Keywords:** pasta, couscous, cooked pasta, cooked couscous, total polyphenols, free polyphenols, bound polyphenols

## Abstract

Pasta and couscous are popular foods manufactured (in their traditional form) from durum wheat semolina. In recent years, the consumers’ quest for novel, functional, gluten-free, wholegrain foods has prompted the industry to manufacture new pasta and couscous products in which durum wheat has been partially or totally replaced by other vegetable flours. Besides dietary fibre, these raw materials might be an interesting source of phytochemicals. In this work, 16 commercial samples of pasta and four samples of couscous representative of the new products and made of refined and wholegrain flours of different species of cereals, pseudocereals and legumes were analysed for free, hydrolysable bound and total polyphenol content by means of the Folin-Ciocalteu procedure. Analyses were repeated on cooked samples to assess the quantity of polyphenols ingested by the consumers. The raw legume and pseudocereal products had a total polyphenol content higher than most cereal products (up to 1743.4 mg of Gallic Acid Equivalent (GAE) per 100 g dry weight). Wholegrain products had higher contents than refined products. The free fraction underwent up to 46% loss with cooking, probably because of solubility in water. The water absorption of pasta and couscous during cooking was in a ratio of 2:3, resulting in higher dilution of polyphenols in the cooked couscous.

## 1. Introduction

Pasta and couscous are two traditional foods in the Mediterranean area nowadays consumed worldwide. They are manufactured using different technologies, however, in the traditional version they share the same raw material, which is refined durum wheat semolina [1,2].

In industrialized countries including Italy, in the last decade the movement towards healthier eating [3] and the consumers’ quest for novel [4], functional [5], gluten-free (GF) [6] and other -free food, as well as wholegrain food [7] has prompted the pasta and couscous industry to produce and market a number of products where traditional shapes have been maintained but durum wheat has been partially or totally replaced by other cereals, pseudocereals or other flours of vegetable origin, particularly legumes [8,9]. Raw materials of vegetable origin are a source of phytochemicals with promising biological effects. Among them, phenolic compounds have attracted attention as molecules able to improve the wellbeing and longevity of the human population [10]. Their content, bioavailability and efficacy still continue to be largely investigated and are considered a topic of interest in food science and nutrition [11].

In the new kinds of pasta and couscous, polyphenols might represent a group of phytochemicals which can vary greatly from one product to another considering the different raw materials used. Some recent studies focused their attention on the nutritional aspects of some of these new foods, GF in particular, reporting on the macro and micronutrient content of GF products [12,13,14]. However, we did not find any information on the content of polyphenols in this kind of foods.

In the present study, the quantities of free and bound polyphenols were determined in 20 industrial samples (16 of pasta and 4 of couscous) representing the different kinds of new pasta and couscous that can be found on the market nowadays. Moreover, considering that these foods are consumed after cooking in water and that cooking induces great changes in texture, molecule structure, content and availability [15], determinations were repeated also on cooked samples to assess the real quantities of phenolic compounds that are ingested by the consumers. Phenolics are in fact reported to leach into the cooking water. However, in some cases phenolics in cereal products are reported to increase with cooking, since the process can soften the hard structure and break cellular components, allowing an easier extraction from the matrix [15]. The literature reports also that changes of phenolic acids upon cooking could result from oxidative degradation, release of free acids from conjugate forms, and formation of complex structures of phenolic substances from related compounds [15].

## 2. Materials and Methods

### 2.1. Sampling and Sample Preparation

Twenty samples of commercial dry pasta and couscous manufactured with different cereal species (durum wheat, *Triticum durum* Desf; Kamut, *Triticum turanicum* Jakubz; emmer, *Triticum dicoccum* L.; oats, *Avena sativa* L.; rye, *Secale cereale* L., maize, *Zea mays* L.; and rice, *Oryza sativa* L.) with flours possessing different extraction rates (refined semolina, partially wholegrain flour, wholegrain flour), with pseudo-cereals (buckwheat *Fagopyrum esculentum* Moench) and with legumes (lentils *Lens culinaris* Medik., beans *Phaseolus vulgaris* L.; peas, *Pisum sativum* L.) were bought in supermarkets. Sixteen samples were of pasta intentionally selected of the same shape (short, tubular shaped, angle cut), whereas four samples were of couscous. Table 1 illustrates the specifications of the samples and their proximate composition as reported on the package labels, together with moisture (determined in our lab, see method in Section 2.3).

Before analysis, raw pasta samples (100 g) were ground by using in sequence a Bühler MLI 204 laboratory mill (Bühler, Uzwill, Switzerland) and a Cyclotec 1093 Sample Mill (0.5 mm sieve; Foss Tecator, Hillerød, Denmark). Raw couscous samples were milled by means of the Cyclotec mill only.

For the analyses on cooked samples, entire (unmilled) pasta and couscous (80 g) were cooked by following the manufacturer’s instructions and cooking times, in unsalted boiling distilled water. Once cooked, the sample was drained and allowed to cool for 30 min, then weighed and, in the case of pasta, cut into small pieces (<5 mm) by means of a knife. Two aliquots of about 20 g were taken for moisture determination (see method in Section 2.3), whereas the remaining part was frozen at −18 °C, lyophilized (Edwards Pirani 1001 lyophiliser, Edwards, Burgess Hill, UK) and finally milled in a water-refrigerated laboratory mill (Janke and Kunkel, Ika Labortechnik, Staufen, Germany).

### 2.2. Chemicals

Methanol, 96% (*w*/*w*) sulphuric acid, Folin-Ciocalteu reagent and sodium carbonate 20% (*w*/*w*) solution were purchased from Carlo Erba (Milan, Italy). Gallic acid was purchased from Sigma (Saint Louis, MO, USA). Acetone was from VWR International (Radnor, PA, USA). All solvents were of analytical grade and reagents were of the highest available purity.

### 2.3. Analyses

Moisture content was determined on raw, cooked and lyophilised samples according to the International Association for Cereal Science and Technology (ICC) standard method 110/1 (ICC, 2003).

Total polyphenols were analysed in raw and in cooked lyophilised samples as follows. Aqueous-organic extracts (extractable polyphenols) and their residues (nonextractable polyphenols) were isolated as described by Durazzo et al. [16] with slight modifications. Two grams of sample were weighed (in triplicate) and placed into a Pyrex centrifuge tube (Vetroscientifica, Rome, Italy) with 20 mL of methanol/water 50:50 (*v*/*v*) equipped with a magnetic bar. The tubes were left under stirring at room temperature for 1 h, then they were centrifuged at 2500× *g* for 10 min and the supernatant was recovered. 20 mL of acetone/water 70:30 (*v*/*v*) were added in the tube, the extraction and centrifugation steps were repeated and the supernatants were all combined. The three resulting aqueous-organic extracts were directly assayed for total phenolic content (TPC).

The three extraction residues were left overnight in a ventilated oven at 30 °C, then they were reduced to powder with the help of the water-refrigerated laboratory mill and their residual moisture was checked (according to the abovementioned standard method). Hence, the powdered dried residues were hydrolysed in boiling methanol/sulphuric acid according to Hartzfeld et al. [17]. 200 mg of powdered dried residue were placed in a Pyrex tube (200-mm-high, 250-mm-wide) equipped with a magnetic bar and screw cap (the high part of the tube serves as a condenser). 20 mL of methanol and 2 mL of concentrated sulphuric acid were added, then the tube was placed in an aluminium heating block over a magnetic hot plate equipped with a Vertex thermoregulator (Velp, Usmate Velate, Italy) and the cap was loosely screwed. The tube was gently heated until the very beginning of boiling and left at that temperature under stirring for 16 h. After this time, the tube content was transferred into a graduated cylinder (50 mL) and the hydrolysis tube was rinsed with minimum amounts of distilled water. The cylinder was cooled in an ice bath and the pH was adjusted between 2 and 3 under stirring by dropwise addition of 8 M NaOH. The magnetic bar was removed, the volume of the mixture was adjusted to the nearest mark, and then an aliquot of the mixture was transferred into a Pyrex centrifuge tube and centrifuged at 2500× *g* for 10 min. The supernatant was recovered and assayed for TPC.

The TPC was determined in both aqueous-organic extracts and the hydrolysed residues by using the Folin-Ciocalteau procedure [18]. Briefly, appropriate dilutions of extracts or hydrolysed residues were oxidized by means of the Folin-Ciocalteau reagent, the reaction was neutralized with sodium carbonate and absorbance was measured at 760 nm against a blank after 2 h of reaction at room temperature. Gallic acid was used as a standard.

Mean values are reported plus standard deviation (SD) and ranking. The sum of TPC of both fractions was expressed as total polyphenols.

### 2.4. Statistical Analysis

One-way Analysis Of Variance (ANOVA) and *T*-test were performed by means of the Paleontological Statistics Software Package for Education and Data Analysis (PAST), (version 2.17, University of Oslo, Oslo, Norway). Other calculations were performed by means of MS Excel software (Microsoft, Redmond, WA, USA).

## 3. Results

### 3.1. Proximate Composition and Characteristics of Uncooked Pasta and Couscous Samples

The proximate composition together with salt content, energy and cooking time of the 16 commercial pasta samples and the 4 couscous dried samples as reported on their nutritional label on the package, are presented in Table 1.

In the pasta set, for comparison reasons it has been deemed useful to group samples into three subsets based on the raw materials used, namely: (1) cereals with gluten; (2) gluten-free cereals and pseudocereals; and (3) legumes (Table 1). In the couscous group there were not enough samples to adopt the same criterion and moreover, they were all made of cereals except for the buckwheat one. The legume pasta as a group had the highest protein content (22.0–28.9% on fresh weight (f.w.) compared to 7.4–14.3% f.w. for the remaining samples) and the highest dietary fibre content (7.6–14.9% f.w. compared to 3.0–9.0% f.w.). The lipids content in all pasta samples ranged from 1.2 for pasta made with durum wheat semolina to 9.4% f.w. for pasta made with wholegrain oats, whereas the carbohydrates ranged from 42% f.w. for the black lentil pasta to 77% f.w. for maize pasta. The salt content in the cereals-with-gluten pasta ranged from 0.0027–0.005% f.w., it was higher in the gluten-free cereals and pseudocereals samples (0.047–0.05% f.w.) and in the legumes samples (0.02–0.07% f.w.). The energy of all raw pasta samples spanned 329–383 kcal/100 g. As far as cooking times for all pasta samples were concerned they went from 3 to 11 min with differences within the same subset (Table 1).

The couscous samples (Table 1) had similar protein contents (from 11.4% f.w. in buckwheat to 12.6% f.w. in wholegrain durum wheat). Lipid content was slightly higher in the buckwheat product than in the cereal products (3.0% f.w. vs. 1.5–2.2% f.w.). Carbohydrates were lower in the less refined products as expected (from 63.0% f.w. in barley to 73.2% f.w. in buckwheat), whereas conversely dietary fibre was higher (from 8.8 to 11% f.w. compared to 3.8% f.w. for semolina). Salt content ranged from 0 (barley) to 0.025% f.w. (buckwheat) and it was higher in the buckwheat couscous than in the cereal products. Energy spanned 337 (barley)–373 (buckwheat) kcal/100 g, whereas the cooking time was 4–6 min with durum wheat couscous samples being the quickest to cook.

### 3.2. Polyphenols Content in Raw Pasta and Couscous Samples

The polyphenol content found in the raw pasta and couscous samples is illustrated in Table 2. Data are expressed as dry weight. Raw products had moisture of around 10% (from 7.0 to 11.1%, Table 1).

Total polyphenol content ranged from 415.0 to 1743.4 mg of Gallic Acid Equivalent (GAE) per 100 g (dry weight, d.w.) in raw pasta and from 470.0 to 1117.7 mg GAE/100 g (d.w.) in raw couscous (Table 2). The highest total polyphenol content was found in black lentil pasta (1743.4 mg GAE/100g, d.w.), followed by buckwheat couscous and buckwheat pasta, (respectively 1117.7 and 1063.8 mg GAE/100 g, d.w.). Black beans pasta and rye pasta followed (with 781.0 and 705.0 mg GAE/100 g, d.w. respectively), then peas pasta, red lentils pasta and barley couscous (657.1, 631.6 and 624.8 mg GAE/100 g, d.w. respectively). The other cereal-based products had a TPC comprised of between 415.0 and 585.0 mg GAE/100 g (d.w.).

The free fraction ranged from 48.7 to 570.8 mg GAE/100 g (d.w.) in raw pasta (representing the 10.3–51.5% of the total) and from 76.7 to 476.2 mg GAE/100 g (d.w.) in raw couscous (13.3–42.6 of the total). Actually, the free fraction represented less than 15% of the total polyphenol content for pasta made of oats, red lentils, maize, peas and rice; it represented between 17% and 20% for wheat and kamut pasta; between 20% and 25% for emmer and rye pasta, 32.7% for black lentils pasta and roughly half for buckwheat and black bean pasta. Similar proportions were found in the couscous samples (Table 2).

The hydrolysable bound fraction was generally the most abundant, ranging from 344.1 to 1172.6 mg GAE/100 g (d.w.) in raw pasta (representing 48.5–89.7% of the total) and from 389.6 to 641.5 in raw couscous (57.4–86.7% of the total considering the whole set of samples).

### 3.3. Polyphenols Content in Cooked Pasta and Couscous

The quantities of polyphenols (total, free and hydrolysable bound) that were found in the dry matter of cooked pasta and couscous samples are also reported in Table 2. Total polyphenol content ranged from 394.8 to 1833.5 mg GAE/100 g (d.w.) in the cooked pasta samples and from 451.7 to 1062.7 mg GAE/100 g (d.w.) in the cooked couscous samples. Hence, this parameter in the cooked samples substantially follows the same order that in the raw samples, with the cooked legume and pseudocereal products having a higher TPC than the cooked cereal products: it is noted that the wholegrain rye pasta loses up to 14% of its original content with cooking.

The free fraction was clearly reduced in the cooked samples with respect to the raw ones, going from 36.8 to 555.0 mg GAE/100 g (d.w.) in the cooked pasta and from 51.6 to 418.6 mg GAE/100g (d.w.) in the cooked couscous.

The hydrolysable bound fraction did not change much, ranging from 353.4 to 1278.5 mg GAE/100 g (d.w.) in the cooked pasta and from 400.1 to 644.1 mg GAE/100 g (d.w.) in the cooked couscous.

Table 3 illustrates the total, free and bound polyphenol content found in the cooked samples, expressed on fresh weight (f.w.), that is taking into account the fact that both pasta and couscous are highly hydrated with cooking: cooked pasta had up to 50% moisture and cooked couscous about 80%. Total polyphenols ranged from 205.3 to 813.5 mg GAE/100 g (f.w.) in the cooked pasta samples and from 89.9 to 169.3 mg GAE/100 g (f.w.) in the cooked couscous samples. The free fraction was 21.4–246.2 mg GAE/100g (f.w.) in the cooked pasta and 10.3–66.7 mg GAE/100 g (f.w.) in the cooked couscous. Finally, the bound fraction was 176.8–567.2 mg GAE/100 g (f.w.) in the cooked pasta and 79.6–111.2 mg GAE/100g (f.w.) in the cooked couscous.

## 4. Discussion

The proximate composition and hence the nutritional value of both dry pasta and couscous are clearly dependent on the raw materials used for their production (botanical species) and in the case of cereals also on the extraction rate of the flours used. Hence, legume pasta has a higher protein content than cereal pasta, whereas wholegrain pasta or couscous has a higher dietary fibre content that the corresponding non-wholegrain pasta or couscous (Table 1). Moreover, judging from the higher number of different products we could find in the supermarkets under the classification of pasta, we could say that the pasta manufacturing process is more versatile than that of couscous, allowing for the use of a great variety of flours of vegetable origin, whereas in the case of couscous we could find cereals and buckwheat only. The energy value of pasta and couscous is also connected to its composition, the highest value (383 kcal/100 g) having been found in wholegrain oat pasta which also has the highest lipid content (9.4% f.w.) (Table 1).

Pasta and couscous are produced by means of different technologies that can affect the structure, the cooking properties and the nutritional properties of the product [1,2]. Short-shaped pasta is generally produced by extrusion and it is dried under various conditions of temperature/time. As a consequence, durum wheat pasta usually contains a low degree of gelatinized starch, whereas its protein structure can be affected if high-temperature drying is used. The main effects of high-temperature drying are protein denaturation, which strengthens the gluten network, and Maillard reaction. Couscous does not require extrusion, but steaming before drying, and hence it presents a high degree of gelatinized starch, together with a higher capability of water absorption when rehydrated during cooking. On the other hand, pasta and couscous produced by gluten-free cereals, pseudocereals and legumes require appropriate formulations (addition of protein extracts that imitate gluten, thickeners or pre-gelatinized starch) and processing conditions (hydrothermal treatment/cooling that induces starch gelatinization/retrogradation, extrusion cooking, and high temperature drying that induces protein denaturation) to assure the tightness of the gluten-free structure that prevents leaching and break-up during cooking [19]. Therefore, the studied samples present different structures which give origin to different cooking times (Table 1) and different abilities to absorb water during cooking.

The profile of phenolic compounds varies with the plant species and variety; moreover, the amount of phenolics in a plant is related to its growing conditions. Phenolic compounds provide a large variety of functions in plants, in reproduction and growth, in defence mechanisms against pathogens, parasites and predators, and in determining colour [20]. Therefore, they are distributed in different parts of the plant, especially in the external parts, where they are free or associated with a variety of molecules like sugars, carbohydrates, amines, proteins, lipids and other phenolics. Soluble phenolics (the “free” fraction), are localized principally in cellular vacuoles, whereas insoluble phenolics (the “bound” fraction) are principally found in cellular walls, bound to cellular components and especially fibre (pectins, arabinoxylans and other polysaccharides), where they contribute to mechanical resistance, growth and morphogenesis, pathogens/stress response, and play a role in cellular adhesion [21]. With regards to human nutrition, phenolic compounds were mostly regarded as antinutrients in the past, whereas nowadays the extent and the mechanism of their protective action against of a number of human diseases due to their antioxidant properties is being deeply investigated [10,11,20,22].

The phenolic compound classes most frequently found in cereal grains are phenolic acids and flavonoids. Phenolic acids of the cinnamic and benzoic series are present, with ferulic acid being the most common; however, the concentration of the different acids depends on the cereal species. Phenolic acids and flavonoids are found in cereals especially in the cell walls of the aleuronic layer, of the pericarp and of the embryo, whereas they are scarcely present in the endosperm; consequently, flours with a higher extraction rate are richer in polyphenols than refined flours [20].

Pulses mainly contain the phenolic compound classes of tannins, phenolic acids (of the benzoic and cinnamic series) and flavonoids; particularly, the highly pigmented varieties owe their colour to the presence of flavonoids such as flavonol glycosides and anthocyanins and to condensed tannins (proanthocyanidins) [22]. Pulses with a high content of polyphenols also show a high antioxidant activity. Some pulse species may contain high levels of free polyphenols. Within pulses, lentils contain the highest amounts of compounds of the cited phenolic classes.

The polyphenol classes mostly found in buckwheat are phenolic acids (especially of the cinnamic series and protocatechuic acid) and flavonoids (especially rutin and quercetin). Buckwheat phenolics are most abundant in the outer layers of the seed, which also show a higher antioxidant activity. A peculiarity of buckwheat is that it has a content of free polyphenols equal to that of bound polyphenols, whereas in cereals and in many legumes the bound fraction is generally preponderant [23].

Given the presence of higher levels of polyphenols in the outer layers of grains with respect to the endosperm, it was expected, in raw products of the same species, to find a higher total polyphenol content when there was a higher extraction rate of the raw material. This was actually found in our samples (Table 2, *p* < 0.1 for wheat products and *p* < 0.05 for emmer products), notwithstanding the fact that they were almost certainly not obtained from the same kernels and not even from the same variety. Moreover, a legal definition of “wholemeal” is lacking at present, allowing terms such as “wholemeal” and “partially wholemeal” to be used in product labels without reference to a specific milling method or to a range of composition specified by law or by a recognised standard.

Cooking reduced the free polyphenol fraction with respect to the original content in the majority of samples (*p* < 0.01, Table 2). The reduction was small and not significant in the case of black lentils and red lentils pasta only. Actually, the reduction of 6–9% for buckwheat pasta and black bean pasta and of 12% for buckwheat couscous, although statistically significant, is doubtfully relevant in practice, because of the sensitivity of the method. In the other samples, free TPC was reduced from 16% up to 46% by cooking. This could have more than one explanation: solubilisation in cooking water, sensitivity to heat/oxygen/water treatment, and association with the food matrix, which is modified by cooking. Loss of free TPC in vegetables due to solubilisation in cooking water is reported by the literature [15].

The amount of bound TPC apparently slightly increases with cooking (Table 2) and decreases in rye pasta only. However, the increase is not statistically significant except for in five pasta samples in which it ranges from 9 to 12%. The decrease in rye pasta is also statistically significant and it is of 7%. Fares et al. [24] report no change of free phenolic acids and a strong increase of bound phenolic acids (from 22 to 46%) in cooked with respect to raw durum wheat pasta samples enriched with debranning fractions of wheat and hypothesizes that cooking liberates some bound phenolic acids. However, these authors compare the levels of single phenolic acids as measured by High Performance Liquid Chromatography (HPLC) and the total phenolic acid content as the sum, and not the total polyphenol content obtained by the Folin-Ciocalteu method. In our case, it remains uncertain if the changes that were detected are practically relevant, due to the sensitivity of the Folin-Ciocalteu method.

In foods cooked in water, polyphenols are diluted by the water absorbed during cooking. Table 3 shows that the cooked couscous contained less total polyphenol than equal weights of cooked pasta. This was particularly evident in cooked buckwheat couscous and pasta. The moisture of cooked pasta was about 40–50%, whereas the moisture of cooked couscous was 77–84%, with respect to about 10% of both raw products. Water absorption was higher for couscous, meaning that it has a higher cooking yield than pasta: in fact, pasta could double its raw weight, whereas couscous could triplicate it by cooking. The standard portion recommended by the Italian Society of Human Nutrition is 80 g of raw product for both pasta and couscous [25] since they have a similar nutritional composition. However, in practice, how much raw pasta/couscous people decide to cook is a personal choice based on several factors, amongst which there is also the cooking yield, so that it may easily occur that consumers decide to cook a lower quantity of raw couscous than raw pasta. Given the polyphenol content in the raw product and the cooking yield due to hydration, the amount of product that is actually eaten can make a difference in terms of the polyphenol quantity that is actually ingested.

## 5. Conclusions

At present, it is possible to find on the market many categories of products that are sold under the name of pasta or couscous. However, their nutritional quality can be quite different depending on the raw materials used for their manufacturing. From a nutritional point of view certainly all the studied products can be considered a good source of carbohydrates, however, their richness in other important nutrients (proteins) and bioactive components such as dietary fibre and polyphenols can be valuable and very different. The content of fibre and phenolic substances in cereal products can vary according to the cereal species and to the extraction rate of the flour or meal used to produce them. Interestingly, it appears that pasta and couscous made of certain legume and pseudocereal species can have a higher content of protein, fibre and polyphenols than cereal products (even wholemeal cereal products) and, of note, higher content than the gluten-free cereal products. This aspect could be interesting for those with celiac disease because it has been noted that gluten-free diets are at risk of providing insufficient amounts of fibre, phytochemicals and essential aminoacids [13]. Consumers should become more aware of differences existing amongst foods that are marketed under the same common name (pasta, couscous) in order to be able to choose products that best meet their needs. We must also remember that for foods such as pasta and couscous which are cooked in water, phytochemicals are somehow diluted by the water absorbed during cooking and the dilution was greater for couscous than for pasta.

## Figures and Tables

**Table 1:** Proximate composition, salt, energy and cooking time of commercial samples of short dry pasta and couscous *.

Raw Samples	Moisture (g/100 g)	Proteins (g/100 g (f.w.))	Lipids (g/100 g (f.w.))	Carbohydrates (g/100 g (f.w.))	Dietary Fibre (g/100 g (f.w.))	Salt (g/100 g (f.w.))	Energy (kcal/100 g)	Cooking Time (min)
**PASTA**								
**Cereals with gluten**								
Durum wheat semolina	10.6	11.5	1.2	72.5	3.0	0.005	353	10
Durum wheat semolina and 2% spirulina	8.6	11.5	1.2	72.5	3.0	0.005	353	6
Partially wholegrain durum wheat	11.1	11.4	1.9	68.2	5.9	<0.01	347	10
Wholegrain durum wheat	11.1	11.5	2.0	65.5	9.0	0.005	344	9
Wholegrain kamut	10.5	14.0	2.2	63.0	9.0	0.005	346	10
Partially wholegrain emmer	10.2	11.5	2.0	68.0	6.0	0.005	348	9
Wholegrain emmer	9.9	13.8	3.5	65.1	6.5	0	347	8
Wholegrain oats	7.0	11.8	9.4	58.3	9.0	0.0035	383	5
Wholegrain rye	9.8	10.1	2.2	62.1	-	0.0027	337	6
**Gluten-free cereals and pseudocereals**								
Maize	9.8	7.5	1.8	77.0	1.7	0.05	358	9–11
Wholegrain rice	10.7	7.4	3.4	73.3	3.0	<0.1	359	9–11
Wholegrain buckwheat	10.1	14.3	3.4	66.3	4.2	0.047	361	3–4
**Legumes**								
Red lentils	10.2	26.1	1.7	49.7	7.6	<0.01	334	5–7
Black lentils	9.3	28.9	1.9	42.0	14.9	0.007	331	3
Black beans	9.6	22.0	2.1	48.5	13.8	0.02	329	8–10
Peas	9.2	23.5	1.5	51.7	12.5	0.02	339	3
**COUSCOUS**								
Durum wheat semolina	8.3	12.0	1.5	70.2	3.8	0.005	350	4
Wholegrain durum wheat	9.6	12.6	2.2	64.0	8.8	<0.01	344	4
Barley	9.3	12.0	1.9	63.0	11.0	0	337	5
Buckwheat	8.4	11.4	3.0	73.2	-	0.025	373	6

f.w., fresh weight; -, data missing on the label. * All data except for moisture which was determined in our lab, were copied from the food label.

**Table 2:** Total polyphenol content, free and hydrolysable bound fractions in commercial samples of dry short pasta and raw couscous and in the corresponding cooked samples.

Samples	Total Polyphenols						Free (Aqueo-Organic Extract)						Bound (Hydrolysable Residue)					
	Raw			Cooked		*p*	Raw			Cooked		*p*	Raw			Cooked		*p*
	mg GAE/100 g (d.w.)	SD	Rank ^#^	mg GAE/100 g (d.w.)	SD		mg GAE/100 g (d.w.)	SD	Rank ^#^	mg GAE/100 g (d.w.)	SD		mg GAE/100 g (d.w.)	SD	Rank *	mg GAE/100 g (d.w.)	SD	
**PASTA**																		
**Cereals with gluten**																		
Durum wheat semolina	415.0	6.0	*k*	394.8	14.2	n.s.	70.8	3.2	*i*	41.4	0.3	−42% **	344.1	3.7	*i*	353.4	14.1	n.s.
Durum wheat semolina and 2% spirulina	437.7	8.1	*jk*	457.7	2.9	+5% *	93.2	1.4	*fg*	70.5	1.7	−24% **	344.5	6.7	*i*	387.2	3.3	+12% **
Partially wholegrain durum wheat	505.1	19.0	*ghi*	457.4	10.4	−9% *	89.4	1.9	*fgh*	48.0	0.3	−46% **	415.6	17.9	*efg*	409.5	10.3	n.s.
Wholegrain durum wheat	538.2	14.2	*g*	532.5	27.9	n.s.	86.2	1.9	*ghi*	61.5	1.1	−29% **	451.9	12.5	*de*	471.0	26.9	n.s.
Wholegrain kamut	585.0	15.5	*f*	555.0	8.0	−5% *	98.2	4.1	*f*	63.7	0.3	−35% **	486.8	12.7	*d*	491.3	7.9	n.s.
Partially wholegrain emmer	493.5	2.4	*hi*	472.4	11.2	−4% *	102.8	3.1	*f*	64.1	0.0	−38%**	390.8	1.4	*fgh*	408.3	11.2	n.s.
Wholegrain emmer	523.6	11.1	*gh*	522.0	3.8	n.s.	138.7	12.4	*e*	93.7	3.8	−32% **	384.9	8.7	*gh*	428.3	3.2	+11% **
Wholegrain oats	471.7	8.0	*ij*	456.3	2.8	−3% *	48.7	1.4	*j*	40.7	0.9	−16% **	423.0	6.6	*ef*	415.7	2.2	n.s.
Wholegrain rye	705.0	4.5	*d*	609.2	7.6	−14% **	174.0	2.1	*d*	116.1	1.4	−33% **	531.0	2.6	*c*	493.1	9.0	−7% **
**Gluten-free cereals and pseudocereals**																		
Maize	421.8	8.9	*k*	417.1	14.6	n.s.	54.3	1.6	*j*	36.8	1.1	−32% **	367.4	10.3	*hi*	380.3	15.2	n.s.
Wholegrain rice	497.1	9.0	*ghi*	468.0	15.7	−6% *	71.7	4.2	*i*	53.6	0.2	−25% *	425.5	5.0	*ef*	414.4	15.8	n.s.
Wholegrain buckwheat	1063.8	9.7	*b*	1090.9	22.4	n.s.	490.4	0.9	*b*	460.7	5.5	−6% **	573.4	10.4	*b*	630.1	23.1	+10% *
**Legumes**																		
Red lentils	631.6	16.6	*e*	629.1	3.3	n.s.	75.1	1.6	*hi*	68.9	4.6	n.s.	556.5	17.8	*bc*	560.2	1.7	n.s.
Black lentils	1743.4	9.6	*a*	1833.5	18.0	+5% **	570.8	6.0	*a*	555.0	6.6	n.s.	1172.6	6.8	*a*	1278.5	11.5	+9% **
Black beans	781.0	10.1	*c*	778.0	14.2	n.s.	402.1	3.9	*c*	365.5	2.0	−9% **	378.9	9.5	*ghi*	412.5	13.4	+9% *
Peas	657.1	22.0	*e*	669.1	17.2	n.s.	88.5	1.5	*fgh*	74.1	1.1	−16% **	568.6	21.8	*bc*	594.9	17.3	n.s.
**COUSCOUS**																		
Durum wheat semolina	470.0	6.2	*d*	451.7	4.1	−4% *	80.4	3.0	*c*	51.6	0.6	−36% **	389.6	9.2	*c*	400.1	4.7	n.s.
Wholegrain durum wheat	574.5	4.0	*c*	563.2	7.5	n.s.	76.7	0.1	*c*	62.2	0.4	−19% **	497.8	4.0	*b*	501.0	7.3	n.s.
Barley	624.8	7.2	*b*	616.7	11.4	n.s.	141.5	3.0	*a*	118.0	1.5	−17% **	483.4	10.0	*b*	498.7	11.9	n.s.
Buckwheat	1117.7	9.3	*a*	1062.7	8.7	−5% **	476.2	2.4	*b*	418.6	9.0	−12% **	641.5	10.5	*a*	644.1	11.7	n.s.

GAE, Gallic Acid Equivalent; d.w., dry weight; SD, standard deviation; *p*, percent difference between values of raw and cooked samples and level of statistical significance: * *p* < 0.05; ** significant, *p* < 0.01; n.s., not significant. ^#^ Equal letters correspond to a difference below the least significant difference after one-way Analysis Of Variance (ANOVA). ANOVA was performed separately on the pasta and the couscous sample sets.

**Table 3:** Total polyphenol content, and free and hydrolysable bound fractions in cooked commercial samples of pasta and couscous.

Cooked Samples	Total Polyphenols			Free (Aqueo-Organic Extract)			Bound (Hydrolysable Residue)		
	mg GAE/100 g (f.w.)	SD	Rank ^#^	mg GAE/100 g (f.w.)	SD	Rank ^#^	mg GAE/100 g (f.w.)	SD	Rank ^#^
**PASTA**									
**Cereals with gluten**									
Durum wheat semolina	205.3	7.4	*k*	21.5	0.2	*h*	183.8	7.3	*ij*
Durum wheat semolina and 2% spirulina	225.2	1.4	*jk*	34.7	0.8	*f*	190.5	1.6	*i*
Partially wholegrain durum wheat	231.5	5.3	*ij*	24.3	0.1	*h*	207.2	5.2	*hi*
Wholegrain durum wheat	300.1	15.7	*de*	34.7	0.6	*f*	265.4	15.2	*de*
Wholegrain kamut	284.7	4.1	*ef*	32.7	0.2	*fg*	252.0	4.1	*ef*
Partially wholegrain emmer	247.0	5.9	*hij*	33.5	0.0	*fg*	213.5	5.9	*ghi*
Wholegrain emmer	276.6	2.0	*efg*	49.7	2.0	*e*	227.0	1.7	*fgh*
Wholegrain oats	261.9	1.6	*fgh*	23.4	0.5	*h*	238.6	1.3	*fg*
Wholegrain rye	327.7	4.1	*c*	62.4	0.7	*d*	265.3	4.8	*de*
**Gluten-free cereals and pseudocereals**									
Maize	242.3	8.5	*hij*	21.4	0.6	*h*	221.0	8.8	*gh*
Wholegrain rice	255.5	8.6	*ghi*	29.3	0.1	*g*	226.2	8.6	*gh*
Wholegrain buckwheat	550.5	11.3	*b*	232.5	2.8	*b*	318.0	11.7	*b*
**Legumes**									
Red lentils	323.3	1.7	*cd*	35.4	2.4	*f*	287.9	0.9	*cd*
Black lentils	813.5	8.0	*a*	246.2	2.9	*a*	567.2	5.1	*a*
Black beans	333.5	6.1	*c*	156.7	0.8	*c*	176.8	5.8	*j*
Peas	333.6	8.6	*c*	37.0	0.6	*f*	296.6	8.6	*bc*
**COUSCOUS**									
Durum wheat semolina	89.9	0.8	*d*	10.3	0.1	*d*	79.6	0.9	*c*
Wholegrain durum wheat	116.6	1.5	*c*	12.9	0.1	*c*	103.7	1.5	*b*
Barley	137.5	2.5	*b*	26.3	0.3	*b*	111.2	2.7	*a*
Buckwheat	169.3	1.4	*a*	66.7	1.4	*a*	102.6	1.9	*b*

GAE, Gallic Acid Equivalent; f.w., fresh weight; SD, Standard Deviation. ^#^ Equal letters correspond to a difference below the least significant difference after one-way Analysis Of Variance (ANOVA). ANOVA was performed separately on the pasta and the couscous sample sets.
